# Introduction to Medical Jurisprudence

**Published:** 1902-12

**Authors:** 


**Introduction to Medical Jurisprudence.** By William McCallin, M.B. Pp. vii., 151. London: Baillière, Tindall & Cox. 1901.

This volume may be useful as a first introduction to larger works, or to enable a student to revise what he has read; but taken alone it can give very little instruction. It is written in too good a style for an epitome, and what it says is accurate, and the work of a man who knows his subject. Moreover, it has some paragraphs which are too often absent in larger works: such as that on the legal status of the medical practitioner, and on gifts to medical men; but a hundred and fifty pages of good-sized print cannot possibly suffice to expound the subject matter. No one without other knowledge could understand or act upon many of the statements; for instance, what is "full apprehension and actual danger of death, which ensues," in the definition of a dying declaration? It is quite true, but useless for guidance. The courts have actually rejected a declaration, which stated that the person had "no hope of recovery at present," as insufficent. Moreover, there are practically no references, so that it is impossible to refer to the statute or authority on which such statements are based. We cannot help regretting that so able a writer has not found time to make a better book.

